# Flavonoids and Phenolic Acids Content in Cultivation and Wild Collection of European Cranberry Bush *Viburnum opulus* L.

**DOI:** 10.3390/molecules28052285

**Published:** 2023-03-01

**Authors:** Sylwia Goławska, Iwona Łukasik, Adrian Arkadiusz Chojnacki, Grzegorz Chrzanowski

**Affiliations:** 1 Institute of Biological Sciences, Faculty of Sciences, Siedlce University of Natural Sciences and Humanities, Prusa 14, 08-110 Siedlce, Poland; 2 Department of Biotechnology, Institute of Biology and Biotechnology, University of Rzeszow, 8B Zelwerowicza Street, 35-601 Rzeszow, Poland

**Keywords:** phenolic acids, flavonoids, *Viburnum opulus*, wild plants, garden variety

## Abstract

Guelder rose (*Viburnum opulus* L.) is known for its health benefits. *V. opulus* contains phenolic compounds (flavonoids and phenolic acids), a group of plant metabolites with wide biological activities. They are good sources of natural antioxidants in human diets owing to their prevention of the oxidative damage responsible for many diseases. In recent years, observations have shown that an increase in temperature can change the quality of plant tissues. So far, little research has addressed the problem of the common impact of temperature and place of occurrence. Towards a better understanding of phenolics concentration that could indicate their potentials as therapeutic agents and towards predicting and controlling the quality of medicinal plants, the aim of this study was to compare phenolic acids and flavonoids content in the leaves of cultivation and wild collection *V. opulus*, and to examine the impacts of temperature and place of occurrence on their content and composition. Total phenolics were determined using the spectrophotometric method. Phenolic composition of *V. opulus* was determined using high-performance liquid chromatography (HPLC). The following hydroxybenzoic acids there were identified: gallic, p-hydroxybenzoic, syringic, salicylic, benzoic, as well as hydroxycinnamic acids: chlorogenic, caffeic, p-coumaric, ferulic, o-coumaric and t-cinnamic. The analysis of extracts from *V. opulus* leaves has indicated the presence of the following flavonoids: flavanols: (+)-catechin and (−)-epicatechin; flavonols: quercetin, rutin, kaempferol, myricetin; and flavones: luteolin, apigenin and chrysin. The dominant phenolic acids were p-coumaric and gallic acids. The major flavonoids found in *V. opulus* leaves were myricetin and kaempferol. Temperature and plant location affected the concentration of tested phenolic compounds. The present study shows the potential of naturally grown and wild *V. opulus* for the human.

## 1. Introduction

Plants are sources of natural bioactive compounds, secondary metabolites and antioxidants [1,2]. Bioactive components produced are stored in plant leaves and most of them are commercially important, especially phenolic acids and flavonoids [3]. Phenolic compounds are important in plants and the human health. Phenolic acids and flavonoids possess wide biological activities: antiulcer, anti-inflammatory, cytotoxic, antispasmodic and antidepressant [4,5,6,7]. These compounds also have antioxidant and anticarcinogenic effects [8,9].

The European cranberry bush, *Viburnum opulus* L., commonly known as the guelder rose or highbush cranberry, is one of the most widespread shrub species. Guelder rose grows under different climatic conditions. It is widely cultivated in gardens in many countries in Europe and Asia [10]. *V. opulus* is a valuable decorative, medicinal and food plant [11,12,13]. Mainly, it is cultivated as an ornamental plant, but *V. opulus* L. is also known for its health benefits [14]. They result from the presence of bioactive components in the plant. Phytochemical studies on this species have shown the presence of different natural compounds such as phenolic compounds, vitamin C, carotenoids, triterpenes, iridoids, essential oils, saponins and dietary fiber [12,14,15,16]. *V. opulus* fruits, fruit juices, flowers, leaves, branches and brank extracts are rich with biologically active substances known for their antioxidative properties and were used in traditional and folk medicine [16,17,18,19,20,21,22,23]. They have been used to treat a wide range of illnesses, including bleeding, heart disease, high blood pressure, coughs and cold, neurosis and diabetes [11,24,25,26]. Studies also show that some dangerous chemicals such as coumarin that can be dangerous to human health [27]. The results of in vitro studies indicate the antimicrobial potential of *V. opulus*, too. *V. opulus* has been demonstrated to possess antibacterial effects against several pathogenic Gram-positive and Gram-negative bacteria. The juice of *V. opulus* fruits strongly inhibited the growth of a wide range of human pathogenic bacteria, both Gram-negative (*Salmonella typhimurium* and *S. agona*) and Gram-positive (*Staphylococcus aureus*, *Lysteria monocytogenes*, and *Enterococcus faecalis*) organisms. Conversely, the yeasts *Debaryomyces hansenii* and *Torulaspora delbrueckii* showed complete resistance to the fruit juice, whereas a low sensitivity was demonstrated by *Trichosporon cutaneum*, *Kluyveromyces marxianus* var. *lactis*, *Saccharomyces cerevisiae*, *S. cerevisiae* 12R and *Candida parapsilosis* [25]. On the other hand, Yilmaz et al. [20] tested the isolated essential oils of *V. opulus*, *V. lantana* and *V. orientala* for antimicrobial activity against the bacteria *Escherichia coli*, *Klebsiella pneumoniae*, *Pseudomonas aeruginosa*, *Enterococcus faecalis*, *Staphylococcus aureus*, *Bacillus cereus* and the fungus *Candida tropicalis*. No activity was observed against all the test microorganisms for *V. lantana* and *V. opulus*. Moreover, the essential oil of the *V. orientale* showed weak antibacterial activity against Gram-positive bacteria.

The levels of bioactive compounds vary between fruit species, genotypes and different environmental conditions (temperature, soil, water, etc.) [28,29,30,31]. Wild edible fruits show a rich biodiversity, so they may constitute the basis for human survival and economic well-being because they can be harvested from forests and marginal lands of rural areas without commercial cultivation [32]. They represent cheap but quality nutrition for the population in both urban and rural areas [33]. Several diverse raw or processed products can be obtained from wild edible fruits of *V. opulus*. They can support household subsistence and also generate income for people. This situation, together with important role in human health and nutrition as sources of vitamins, minerals, antioxidants, dietary fiber and phytonutrients (plant-derived micronutrients), is the reason for the study of those valuable plants [34,35]. Bioactive compounds of *V. opulus* can help the human body to be fit, rejuvenate, and stay free of diseases [36,37,38].

In Poland, wild *V. opulus* plants are common, are varieties grown in gardens are also encountered. However, these plants and their fruits are still unknown or insufficiently exploited in Poland despite their nutritional value. The content and elementary chemical composition of flowers, bark and fruits of *V. opulus* were previously analyzed by others [14,39,40,41,42,43]. Known components of bark and fruits of *V. opulus* L. are catechine, tannins, coumarins (scopoletin, esculetin), flavonoids (astragalin, kaempferol, quercetin, amentoflavon), sterols and triterpenes [12,40,44,45,46].

Although there have been some detailed reports on the bioactive and biochemical characteristics of *V. opulus* grown in different parts of the world, there have not been many studies in Poland. Moreover, there are no studies on the effect of temperature on the content of phenolic compounds in *V. opulus*, which is important due to global warming. Observations in recent years have shown that climate change can affect plants. An increase in temperature can, among other factors, cause a reduction in plant growth, leaf elongation, a disturbance in the process of photosynthesis, the translocation of sugars and changes in the quality of plant tissues [47,48,49]. In particular, no research has been conducted on the chemical content of *V. opulus* plants grown in the wild and in gardens. The chemical composition of *V. opulus* leaves, for which health-promoting effects have also been demonstrated, has seen significantly less investigation so far and little is known about their chemical characteristic. Therefore, it would be useful to have better knowledge of phenol compounds concentration, which could indicate how they control the quality of the plants, all in service of isolating the components for a number of pharmaceutical compounds. To draw attention to the possibility of using leaves of the *V. opulus* for humans, in different commercial products —for examples, cosmetics functional foods or pharmaceutics—the objective was to evaluate and compare the concentrations of phenolic acids and flavonoids in the leaves of *V. opulus* growing in the wild, as well as the garden variety. We hypothesized that temperature and place of occurrence will affect some metabolites content in *V. opulus* shrubs. The impact of temperature and place of occurrence on plant metabolites content has not been studied for *V. opulus.* Therefore, this study aimed to determine how temperature and place of occurrence changes the secondary metabolites in *V. opulus* tissues.

## 2. Results

### 2.1. Total Phenols

The concentration of total phenols depends on analyzed parameters (GLM; F_21,146_ = 6.50; *p* < 0.001; R^2^ = 0.48). The obtained results were significantly influenced by the place and by the survey number, while the temperature was not important (Table 1).

**Figure 1:**
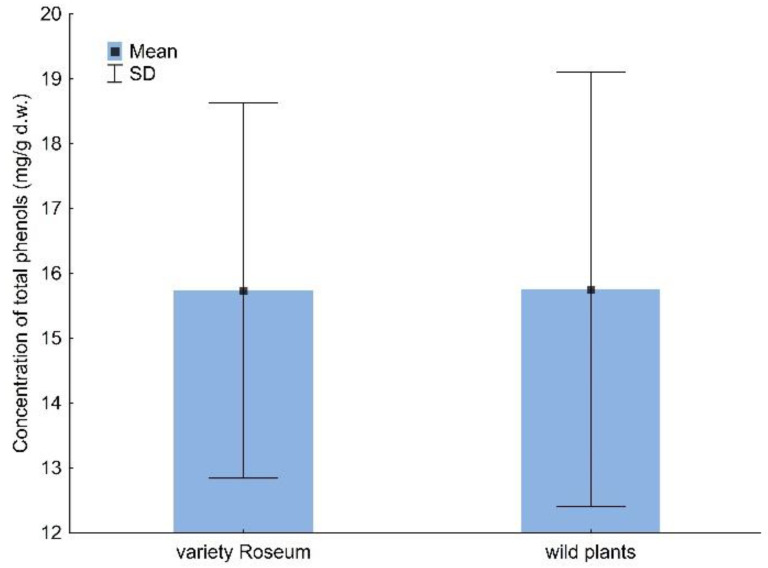
The concentration (mg/g d.w.) of total phenols in *V. opulus* variety Roseum and wild plants.

The average concentration of total phenols in studied *V. opulus* plants ranged between 15.73 and 15.75 mg/g d.w for variety Roseum and wild plants (Figure 1). There was significant interaction between survey number and place for total phenols (Table 1).

### 2.2. Total Flavonoids

**Figure 2:**
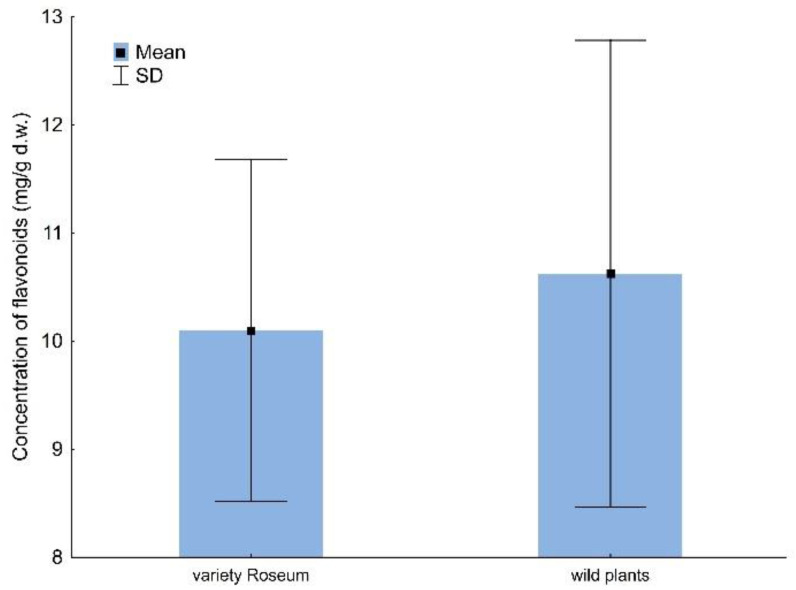
The concentration (mg/g d.w.) of total flavonoids in *V. opulus* variety Roseum and wild plants.

The concentration of total flavonoids depends on analyzed parameters (GLM; F_21,146_ = 19.48; *p* < 0.001; R^2^ = 0.74). The obtained results were significantly influenced by survey number, while the temperature and place were not important (Table 1). The average concentration of total flavonoids in studied *V. opulus* plants ranged between 10.62 and 10.10 mg/g d.w for wild and garden plants (Figure 2). There was a significant interaction between survey number and place for total flavonoids (Table 1).

### 2.3. Phenolic Acids

**Figure 3:**
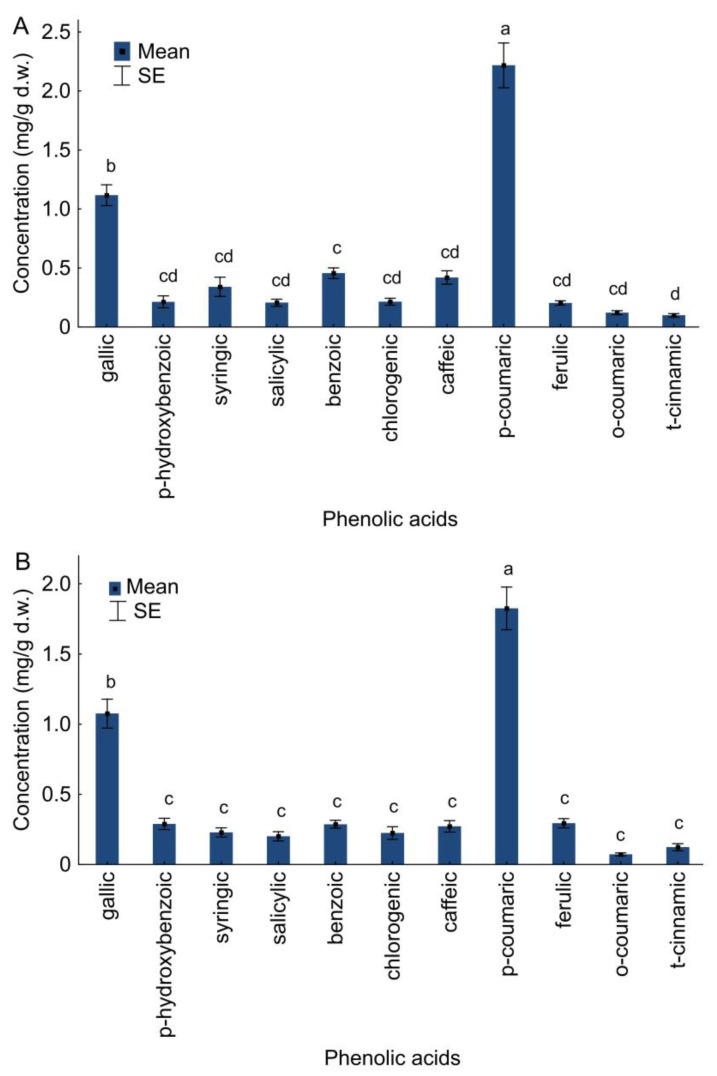
The concentration (mg/g d.w.) of individual phenolic acids in *V. opulus* variety Roseum (**A**) and wild plants (**B**). Different letters denote significant differences (one–way ANOVA; Tukey’s test; *p* < 0.05).

Garden variety and wild varieties of guelder rose shrubs had similar phenolic acid profiles. On the basis of the absorption spectra of the chromatograms, eleven phenolic acids—five hydroxybenzoic acids (gallic, p-hydroxybenzoic, syringic, salicylic, benzoic) and six hydroxycinnamic acids (chlorogenic, caffeic, p-coumaric, ferulic, o-coumaric and t-cinnamic)—were identified. There were differences in the content of phenolic acids in *V. opulus* tissues variety Roseum (one-way ANOVA, F_10,121_ = 72.90, *p* < 0.001) and wild plants (one-way ANOVA, F_10,121_ = 71.88, *p* < 0.001). It was shown that p-coumaric and gallic acids were dominant in *V. opulus* plants. Concentration of gallic acid was 1.12 mg/g d.w. for variety Roseum and 1.08 mg/g d.w. for *V. opulus* plants grown in the wild. Concentration of p-coumaric was 2.22 mg/g d.w. and 1.83 mg/g d.w. for *V. opulus* grown in gardens and the wild, respectively (Figure 3). The content of other hydroxybenzoic and hydroxycinnamic acids was low and similar (Figure 3).

The concentration of three hydroxybenzoic acids depends on the analyzed factors: p-hydroxybenzoic acid (GLM; F_4,19_ = 11.58; *p* < 0.001; R^2^ = 0.71), salicylic acid (GLM; F_4,19_ = 3.27; *p* = 0.034; R^2^ = 0.41) and benzoic acid (GLM; F_4,19_ = 2.93; *p* = 0.048; R^2^ = 0.38). The obtained results for benzoic and p-hydroxybenzoic acids were significantly influenced by the place, while the temperature and survey number were not important (Table 2).

Compared to wild plants, in tissues of *V. opulus* variety Roseum, the content of benzoic acid was higher and p-hydroxybenzoic acid was lower (Figure 3). There was no effect of the analyzed factors on the concentration of other identified hydroxybenzoic acids: gallic (GLM; F_4,19_ = 2.28; *p* = 0.098; R^2^ = 0.32) and syringic (GLM; F_4,19_ = 1.89; *p* = 0.154; R^2^ = 0.28). The analyzed factors also affected the concentration of five hydroxycinnamic acids: chlorogenic (GLM; F_4,19_ = 11.71; *p* < 0.001; R^2^ = 0.71), caffeic (GLM; F_4,19_ = 7.73; *p* < 0.001; R^2^ = 0.62), p-coumaric (GLM; F_4,19_ = 7.78; *p* < 0.001; R^2^ = 0.62), ferulic (GLM; F_4,19_ = 9.68; *p* = 0.001; R^2^ = 0.67) and o-coumaric (GLM; F_4,19_ = 11.56; *p* < 0.001; R^2^ = 0.70).

For caffeic, p-coumaric, ferulic and o-coumaric acids, the obtained results were significantly influenced by place (Table 3). Caffeic, p-coumaric and o-coumaric acids in higher concentrations and ferulic acid in a lower concentration were found in *V. opulus* variety Roseum (Figure 3). Temperature was important for chlorogenic and ferulic acids. Survey number was not important (Table 3). There was no effect of the analyzed factors on the concentration of t-cinnamic acid (GLM; F_4,19_ = 1.30; *p* = 0.306; R^2^ = 0.21) (Table 3). There was a significant interaction between survey number and place for salicylic, chlorogenic and ferulic acids (Table 2 and Table 3).

### 2.4. Flavonoids

**Figure 4:**
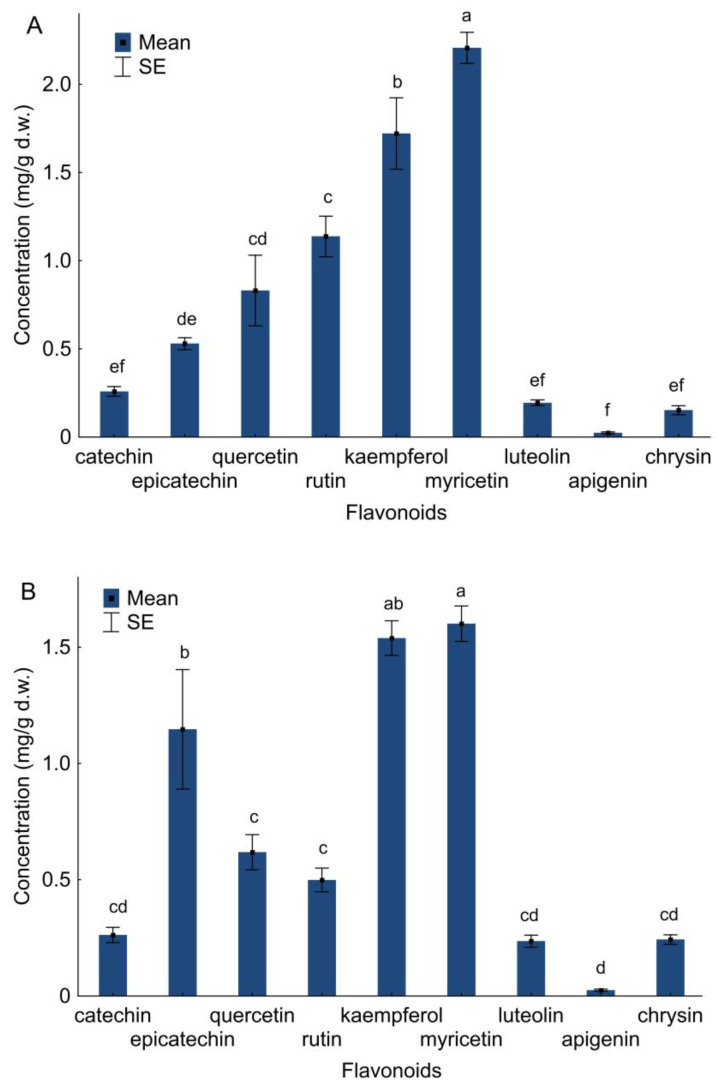
The concentration of individual flavonoids in *V. opulus* variety Roseum (**A**) and wild plants (**B**) (means ± SE; *n* = 12). Different letters denote significant differences (one–way ANOVA; Tukey’s test; *p* < 0.05).

Garden variety and wild guelder rose shrubs had similar flavonoids profiles. Nine flavonoids—two flavanols ((+)-catechin and (−)-epicatechin), four flavonols (quercetin, rutin, kaempferol, myricetin) and three flavones (luteolin, apigenin and chrysin)—were identified in garden variety and wild guelder rose shrubs. There were differences in the content of flavonoids in *V. opulus* tissues variety Roseum (one-way ANOVA, F_8,99_ = 50.20, *p* < 0.001) and wild plants (one-way ANOVA, F_8,99_ = 36.28, *p* < 0.001). It was shown that the compounds myricetin (variety Roseum: 2.21 mg/g d.w.; wild plants: 1.60 mg/g d.w.) and kaempferol (variety Roseum: 1.72 mg/g d.w.; wild plants: 1.54 mg/g d.w.) were dominant in *V. opulus* plants and (−)-epicatechin (1.15 mg/g d.w.) in *V. opulus* wild plants (Figure 4). The content of other flavonoids was low (Figure 4).

The concentration of two flavanols (+)-catechin (GLM; F_4,19_ = 4.73; *p* = 0.008; R^2^ = 0.50) and (−)-epicatechin (GLM; F_4,19_ = 278.36; *p* < 0.001; R^2^ = 0.98) depends on the analyzed factors. For catechin, the obtained result was significantly influenced by the survey number, while the place and temperature were not important; epicatechin was influenced by place and survey number (Table 4). Concentration of (+)-catechin was similar and was 0.26 mg/g d.w. for variety Roseum and wild-grown plants. (−)-epicatechin in higher concentrations was found in *V. opulus* wild plants (Figure 4).

The concentration of four flavonols depends on the analyzed factors: quercetin (GLM; F_4,19_ = 96.82; *p* < 0.001; R^2^ = 0.95), rutin (GLM; F_4,19_ = 123.37; *p* < 0.001; R^2^ = 0.96), kaempferol (GLM; F_4,19_ = 18.73; *p* < 0.001; R^2^ = 0.80) and myricetin (GLM; F_4,19_ = 15.73; *p* < 0.001; R^2^ = 0.77). For quercetin, rutin and myricetin, the result was influenced by the place; for quercetin by survey number was also relevant (Table 5). The concentrations of these flavonols were recorded higher for *V. opulus* grown in gardens. The concentration of kaempferol was similar in studied plants (Figure 4).

The analyzed factors also affected the concentration of chrysin (GLM; F_4,19_ = 14.96; *p* < 0.001; R^2^ = 0.76). For chrysin, the obtained results were significantly influenced by all studied factors (Table 6). The concentration of chrysin was higher for wild plants of *V. opulus* (Figure 4). There was no effect of the analyzed factors on the concentration of the other identified flavones: luteolin (GLM; F_4,19_ = 1.57; *p* = 0.222; R^2^ = 0.25) and apigenin (GLM; F_4,19_ = 1.48; *p* = 0.249; R^2^ = 0.24) (Table 6). The concentrations of apigenin and luteolin in variety Roseum and wild plants were similar (Figure 4). There was a significant interaction between survey number and place for analyzed flavonoids, with two exceptions: luteolin and apigenin (Table 6).

## 3. Discussion

Formerly, wild plants and animals were the sole dietary components for hunter–gatherer and forager cultures. Today, every ecosystem has been amended so that plants and animals can be used as food, fiber, fodder, medicines, traps and weapons, but wild plants remain key to many communities [35]. The literature on vulnerability, food security and ecosystem services has tended to emphasize cultivated foods [50]. However, our foods derived from wild plants are an important part of the global food basket. So, the importance and values of wild plants are just as important as those grown in our gardens.

*Viburnum opulus* is common in natural habitats in Europe, some regions of North Africa, Asia and central Russia [16,17,18,19,20,21,22,23,24,25,26,27,28,29,30,31,32,33,34,35,36,37,38,39,40,41,42,43,44,45,46,47,48,49,50,51]. It is a valuable decorative, medicinal and food plant. It is the very popular in Europe and also readily grown in gardens. Interest is *V. opulus* plants also stems from their health benefits, which have to do with the presence of bioactive components, especially phenolic compounds, vitamin C, carotenoids, iridoids and essential oils, among others [12,14,15,16,43,52].

The chemical content in *V. opulus* fruits, flowers and bark was previously analyzed by others [14,39,40,41,42], who found that the content of phenolic compounds in different morphological parts of *viburnum* varied. However, there are very few reports on the basic chemical composition—especially with respect to phenolic compounds—of *V. opulus* leaves. The obtained results for *viburnum* leaves showed that the content of total phenols was in the range of 10.73–10.75 mg/g d.w. for wild plants and variety Roseum, respectively. Total phenols content depends on the survey number and place of cultivation. According to Polka et al. [43], the content of total phenolics in *V. opulus* flowers, bark and fruits was higher, and it was in the range of 3.51–3.98 g/100 g d.w. *V. opulus* bark was characterized by a higher level of total phenolics compared to the fruit and flowers [45]. For comparison, the content of phenolics in *V. opulus* fresh fruits from the Czech Republic was estimated at 0.68–0.83 g/100 g f.w., from Russia, 0.40–0.73 g/100 g f.w., from Turkey, 0.62–0.99 g/100 g f.w. and from Lithuania, 0.75–1.46 g/100 g f.w. [12,14,16,42]. The results regarding the content of given compounds obtained by us are lower compared to those obtained by others. Our results showed that the content of flavonoids in *V. opulus* leaves was in the range of 10.10–10.62 mg/g d.w. for wild plants and variety Roseum, respectively, and flavonoids content depends on the survey number; it does not depend on the place of cultivation. Total flavonoids in *V. opulus* fruits were higher—in the range 187–489 g/100 g f.w. [43]. It is related to the color of the fruit; it has a red skin color due to the presence of anthocyanins and carotenoids. Proanthocyanidins are quantitatively significant components of the fresh *V. opulus* fruits and account for over 50% of total phenolics [12]. In Polka et al.’s [43] study, total proanthocyanidins in *V. opulus* tested products varied from 0.22 in flowers to 1.03 g/100 g d.w. in bark, and accounted for 6.3% of total phenolics in flowers, 13.9% in fruits and 25.9% in bark. Turek and Cisowski [53] reported greater total flavonoids content (1032 mg of (+)-catechin equivalents per 100 g of f.w.) in the seeds of *V. opulus*. Polka and Podsędek [43] determined the concentration of total flavonoids in bark and flowers at the level of 2250 mg and 1670 mg of (+)-catechin equivalents per 100 g of f.w., respectively. In Velioglu et al. [11] and Erylimaz et al.’s [54] study, total flavonoids in *V. opulus* fruit were between 0.20 g–0.49 g of rutin equivalents per 100 g f.w., according to a colorimetric assay, and in Akbulut et al.’s [55] study, from 0.004 to 0.255 g/100 g f.w., according to the HPLC method. In Polka et al.’s [43] study, total flavonoids varied from 1.67 in flowers to 2.25 g (+)–catechine quivalents/100 g d.w. in bark, and they accounted for 47.6, 53.9 and 56.5% of total phenolics in *V. opulus* flowers, fruits and bark, respectively. Ersoy et al. [42] showed that flavonoids accounted for 27.3–37.4% of the total polyphenol content in fresh *V. opulus* fruits. Çam et al. [39] found that seeds contain 3.5–6.8-fold more phenolics and flavonoids than fruit and are a better source of these compounds.

Data on the composition of individual phenolic compounds are very important. They have great diversity, which suggest their function. Research on the qualitative composition of phenolic compounds in *V. opulus* organs, especially leaves, is rare. Polka and Podsędek [45] showed the presence of hydroxycinnamic acids (chlorogenic, neochlorogenic and cryptochlorogenic), flavanols (catechin, procyanidin B1), flavonols (quercetin 3-rutinoside, quercetin 3-glucoside, isorhamnetin and isorhamnetin 3-glucoside) in *V. opulus* flowers, and the presence of flavanols (catechin, epicatechin, procyanidin B1 and B2) and hydroxycinnamic acids (chlorogenic, neochlorogenic, cryptochlorogenic p-coumaric) in bark. In our study, the leaves of *V. opulus* were characterized by the variation of the individual phenolic compounds tested. In the present study, in variety Roseum and wild guelder rose shrubs, we determined phenolic acids such as hydroxybenzoic acids (gallic, p-hydroxybenzoic, syringic, salicylic, benzoic) and hydroxycinnamic acids (chlorogenic, caffeic, p-coumaric, ferulic, o-coumaric and t-cinnamic), and three classes of flavonoids: flavanols ((+)-catechin and (−)-epicatechin), flavonols (quercetin, rutin, kaempferol, myricetin) and flavones (luteolin, apigenin and chrysin). Similar tendency was revealed by others. Turek and Cisowski [53] echoing in *V. opulus* bark our research on leaves, showed the presence of chlorogenic, gallic, caffeic, ferulic, syringic and p-coumaric acids. Moreover, 4-hydroxybenzoic, protocatechuic, 3,4-dixydroxyphenylacetic, 3,4,5-trimetoxybenzoic, homogentisic and ellagic acids were found. In our study, we did not detect these compounds in the leaves. Just like us, Altun and Yilmaz [56], in *V. opulus* leaves and branches, showed chlorogenic acid and salicin. In fruits, leaves, sprouts and steams, iridoids have been found, also [12,57,58]. In *V. opulus* fruits and fruit juice, the presence of hydroxybenzoic (e.g., gallic, vanillic and syringic) and hydroxycinnamic (e.g., chlorogenic, caffeic, coumaric, ferulic) acids, flavanols (e.g., catechin, epicatechin, procyanidin), flavonols (e.g., quercetin) and anthocyanins (e.g., cyanidin) has been shown, and the differences in phenolic composition between the studied *V. opulus* fruit genotypes have been demonstrated. [11,12,40]. In our study, we determined p-coumaric, gallic acids and certain flavonoids (myricetin, kaempferol, (−)-epicatechin and rutin) as the dominant phenolic compounds in *V. opulus* leaves. The literature indicates qualitative and quantitative differences in the content of phenols in different parts of *viburnum* obtained by researchers. Andreeva et al. [17], in bark extract, as we do in our research, reported the presence of caffeic, chlorogenic, p-hydroxybenzoic and gallic acids. Polka et al. [43], in *V. opulus* fruits and flowers, showed hydroxycinnamic acids as the dominated phenolics (fruits 763.32 mg/100 g f.w.; flowers 1559.42 mg/100 g f.w.), and, in bark, flavanols (1712.55 mg/100 g f.w.). Flavonols have not been found in the bark by Polka et al. [43]. in our research Chlorogenic acid was found to be the dominant compound in flowers (1535 mg/100 g f.w.) and fruits (752 mg/100 g f.w.), and (+)-catechin (1062 mg/100 g f.w.) in bark. According to Perova et al. [12], chlorogenic acid was the main compound of fruits. Similar results were obtained by Velioglu et al. [11] who, as the main ingredient of *viburnum* fruit, indicated chlorogenic acids (204 mg/100 g f.w.) and (+)-catechin (29 mg/100 g f.w.). On the other hand, Özrenk et al. [40] showed (+)-catechin (28–35 mg/100 g f.w.) and gallic acid (11–12 mg/100 g f.w.) to be the dominant compounds in *viburnum* fruits. In *V. opulus* fruits and fruit juice, they were identified anthocyanins, too [11,12,46]. We did not identify these compounds in our study, which may be related to research conducted on other parts of the plant and the different conditions in the environments in which the plants grew.

Secondary chemicals, such as flavonoids and phenolic acids, are important in plant use. Most pharmaceuticals are based on secondary metabolites to enhance human immunity [59]. Flavonoids constitute a wide range of substances that play a role in protecting biological systems against the harmful effects of oxidative processes on macromolecules such as proteins, lipids and DNA [2,60]. Some of biological activities of phenolic acids are as follows: it increases bile secretion, reduces blood cholesterol and lipid levels and has antimicrobial activity against some strains of bacteria, e.g., *Staphylococcus aureus* [20,25,61]. The antimicrobial properties of quercetin, rutin, caffeic acid, vanillic acid and gallic acid from different wines against pathogens were investigated [62]. The most sensitive bacterium was *Escherichia coli*, and *Flavobacterium* sp. was resistant against all tested phenolic compounds. All wine samples showed antimicrobial properties, and the inhibition increased when the polyphenols concentration of wines increased. Clarified wines were inactive against all bacteria. It indicates that polyphenolic compounds which are responsible for the antimicrobial effects. Hendra et al. [63] reported the antimicrobial activity of kaempferol, quercetin, myricetin, naringin, and rutin against Gram-positive and Gram-negative bacteria. The presence of these compounds might contribute to antimicrobial activity of *P. macrocarpa* fruit. Cushnie and Lamb [60] reported that kaempferol, myricetin, naringin, quercetin and rutin have antimicrobial activity against human pathogenic microorganisms with some mechanisms of action such as inhibition of nucleic acid synthesis, cytoplasmic membrane function and energy metabolisms. Teffo et al. [64] investigated the antimicrobial activity of kaempferols from *Dodonaea viscosa* Jacq. var. *angustifolia* leaf extracts against *Staphylococcus aureus*, *Enterococcus faecalis*, *E. coli* and *Pseudomonas aeruginosa*. Demetzos et al. [65] investigated the antimicrobial activity of myricetin and its derivate against Gram-positive bacteria. It was shown that quercetin and naringin have antimicrobial activity, too [66,67]. On this basis, and from the results obtained, *V. opulus* leaves could be considered as a natural antimicrobial source due to the presence of phenolic compounds. We showed that *V. opulus* has a diverse phytochemical profile, with phenolic acids such as hydroxybenzoic and hydroxycinnamic acid and classes of flavonoids such as flavonols, flavanols and flavones. The huge structural diversity of these compounds significantly affects their properties, so they can play important roles for the human. Phenolic acids and flavonoids possess diverse biological—e.g., for instance, antioxidant [16,17,18] and antimicrobial [20,25,54]—activities.

The concentration of plant metabolites is affected by abiotic factors: temperature, drought, salinity, altitude, light and UV radiation [68,69]. The most important environmental factors affecting the secondary compounds is temperature [70]. Wen et al. [71] showed that increasing temperature often led to an enhancement of phenolic accumulation. On the other hand, Mori et al. [72] revealed that high temperatures repressed anthocyanin accumulation in various plants. The biosynthesis of flavonoids is largely influenced by the length of the day and the temperature, and in the case of phenolic acids, the place of occurrence [73]. In our study, we showed that environmental conditions influence the content and metabolic profile of phenolic compounds. For similar results, see the vegetable research of Sytar et al. [74]. Their studies have shown the accumulation of total phenolics, flavonoids and phenolic acids (benzoic acid derivatives and cinnamic acid derivatives) increased in direct sunlight (high UV radiation, moderate temperature) conditions outdoors, as compared to the greenhouse conditions (low UV radiation, high temperature). Their results show that in the accumulation of flavonoids, anthocyanins and methoxycinnamic acid, the level of UV radiation plays a dominant role, while temperature predominantly influences the accumulation of phenolic acids. Our study took place in natural conditions, but the position of wild *V. opulus* was more shaded and was not exposed to direct sunlight, unlike the variety Roseum which grew in a sunny position. In our research, the effect of temperature on the content of total phenols and flavonoids was not shown, but we found the effect of temperature on the concentration of single compounds. We found that temperature affected apigenin and chrysin composition, and chlorogenic and ferulic acids. However, the place of occurrence had an influence on the content of total phenols; phenolic acids: p-hydroxybenzoic, benzoic, caffeic, p-coumaric, ferulic and o-ciumaric; and flavonoids: epicatechin, quercetin, rutin, myricetin and chrysin. The content of two phenolic acids—p-hydroxybenzoic and ferulic—and two flavonoids—epicatechin and chrysin—was higher in *V. opulus* wild plants. Lancaster et al. [75] investigated the effect of UV-B irradiation at 10 °C and 20 °C on the quercetin glycosides procyanidins, chlorogenic acid and anthocyanin levels in the skin of apples and there were no common effects of UV-B irradiation and temperature across all cultivars. Flavonoids and phenolic acids were variable, depending on cultivar, previous light exposure, temperature and class of flavonoids examined. Barański et al. [76] found that the concentrations of ferulic, p-coumaric and caffeic acids in einkorn and emmer were higher in dry and very warm cultivation years. Similarly, in our study, the concentration of the most studied phenolic acids was higher in the variety Roseum, which grew on a drier and sunnier site compared to the wild plants. On the other hand, Uleberg et al. [77] found that northern clones of bilberry (*Vaccinium myrtillus* L.) showed significantly higher contents of total anthocyanins, all measured anthocyanin derivatives, total phenolics, malic acid and sucrose, and metabolic profiling revealed higher levels of flavanols, hydroxycinnamic acids, quinic acid and carbohydrates at 12 °C.

As we can see, knowing the qualitative and quantitative composition of chemical compounds in plants is important; the concentration of chemicals may reflect the influence of environmental conditions. Temperature and light are important environmental factors that affect chemicals biosynthesis. We studied only the effect of temperature. It was found that temperature and light conditions affected flavonoid composition through the regulation of flavonoid biosynthesis pathway genes [78]. However, the interrelationships between temperature and light effects in flavonoids and other chemical compounds’ biosynthesis have not been fully elucidated at the molecular level. Previous studies, however, have shown that the application of high temperatures may alter the concentration and composition of phenolic compounds of peel extracts and of processed juices derived from citrus fruit [79].

So, it is very important to determine the qualitative composition of phenolic compounds because the structural diversity of phenolics affects their properties. If we are looking for bioactive components with rich and diverse chemical compositions and biological properties, and if we want to use of the most valuable parts of *V. opulus* in different preparations introduced into our bodies, we must know the chemical composition of the plant from which these preparations will be made. We must also take into account the temperature and the place where the plants grow.

## 4. Materials and Methods

### 4.1. Plant Material and Study Area

The research was carried out during two seasons, in 2016 and 2017, on *Viburnum opulus* L. shrubs grown in green areas around Siedlce, Poland (52°12′ N, 22°17′ E). The climate of the area is characterized by an annual mean temperature of 8.7 °C, annual mean relative air humidity of 79% and a total rainfall of 526 mm (https//en.tutiempo.net (accessed on 10 October 2022)). During the experimental period, the weather was typical for the spring in eastern Poland. Leaves collected from fully expanded 1-year side shoots of the *V. opulus* L. shrubs, growing in the wild and the garden variety Roseum, were used in all experiments. Leaves were harvested, freeze-dried, ground and kept in a desiccator in darkness until analyzed. The measurements were conducted in triplicate on freeze-dried leaves of two shrubs for garden variety and two shrubs for wild plants from the end of April/early May to the end of June/early July, every 10 days.

### 4.2. Chemical Analysis

#### 4.2.1. Determination of Individual Phenolic Acids

Phenolic acids were analyzed according to Czerniewicz et al. [80]. Using ultrasonic bath Sonic-6D (PolSonic, Warsaw, Poland), phenolic acids were extracted from plant material (0.5 g) in 25 mL of 80% methanol at 45 °C for 1 h. The obtained extract was centrifuged at 10,000× *g* for 20 min and the supernatant was collected. To the supernatant solid, sodium bicarbonate and water were added to final concentration 5% (*w*/*v*) of salt and 20% (*v*/*v*) of methanol, sonicated at 40 °C for 1 h, left in darkness overnight and then centrifuged. Afterwards, the supernatant was acidified to pH 3.0 with 6 M HCl and sonicated for 30 min. The extract was evaporated under a vacuum at 40 °C (Hei-VAP Precision, Heidolph Instruments GmbH and Co., KG, Schwabach, Germany). Oily residue was suspended in water and applied onto SPE (solid phase extraction) column equilibrated with water. Using a Chromabond C18ec column (Macherey-Nagel GmbH and Co., KG, Düren, Germany), SPE was carried out with a Visiprep™ SPE Vacuum Manifold (Sigma-Aldrich, Poznan, Poland). The column was washed with H_2_O and phenolic acids were eluted with methanol. Using an HPLC isocratic Varian ProStar system, equipped with a ProStar 210 pump, a ProStar 335 Photodiode Array Detector and a Microsorb MV 100- 5C18 column (4.6 × 250 mm, Agilent, Santa Clara, CA, USA) chromatographic separation were carried out. A mixture of methanol and water (25:75) with the addition of 1% (*v*/*v*) acetic acid was the mobile phase. Identification of the phenolic acids was conducted using Varian software (Star Chromatography Workstation ver. 6.41, Aurora and PolyView 2000). Retention times and the UV–Vis spectra of separated compounds were compared with standards obtained from Sigma-Aldrich. As the internal standard, the p-hydroxybenzoic acid was used. The samples were analyzed in triplicate. The quantity of phenolic acids present in each sample was accomplished by comparing their peak area with that of a calibration curve of each standard.

#### 4.2.2. Determination of Individual Flavonoids

Flavonoids were analyzed according to Czerniewicz et al. [80]. After acidic hydrolysis, analysis of flavonoid compounds was performed. We extracted 0.5 g of powdered plant material in 25 mL of methanol, acidified to pH 2.0 with 6 M HCl, and then the extract was subjected to hydrolysis at 80 °C for 3 h. Afterwards, methanol was added to final volume 20 mL and the mixture was sonicated for 30 min and then centrifuged at 10,000× *g* for 20 min. The extract was evaporated under a vacuum at 40 °C, and the dry residue was suspended in water and applied onto SPE Chromabond C18ec column (Macherey-Nagel) equilibrated with water. Flavonoids were eluted with methanol and analyzed on an Altus A-10 HPLC system equipped with a photodiode array detector (PerkinElmer). Separation of flavonoid aglycones was performed using a Microsorb MV 100-5C18 column (4.6 × 250 mm, Agilent). The column temperature was maintained at 30 °C and the injection volume was 20 mL. The mobile phase consisted of 1% H_3_PO_4_ (Solvent A) and 40% acetonitrile in 1% H_3_PO_4_ (Solvent B). The linear gradient elution was used as follows: 0 min, 20% B; 65 min, 85% B; 70 min, 100% B; 75 min, 100% B; 77 min, 20% B. Flavonoids were detected at 280 nm, 320 nm and 370 nm. Identification of separated compounds was specified on the basis of retention time and UV–Vis spectra of commercial standards (Sigma-Aldrich). As internal standard, the flavonol kaempferol was used. The quantification of each compound was accomplished by comparing their peak area with that of a calibration curve of each standard. The samples were analyzed in triplicate. Data were collected and processed using Empower^®^ 3 (Waters Corporation, Milford, MA, USA) software.

#### 4.2.3. Total Phenols Determination

Total phenolics were determined using Folin–Ciocalteau reagent (Sigma-Aldrich, Poznan, Poland) according to Stratil et al. [81]. For this purpose, 0.2 mL of plant extract was mixed with 6.8 mL of H_2_O, then 0.5 mL of Folin–Ciocalteu reagent (diluted with water 1:1) was added. After 3 min of incubation at room temperature in darkness, 1 mL of 20% Na_2_CO_3_ (Sigma-Aldrich, Poznan, Poland) was added and mixed, and the whole was made up to 10 mL with water. The absorbance of the blue complex was measured spectrophotometrically with a UV–Vis spectrophotometer (Hewlett Packard 8453) at 725 nm, and phenol content was appointed with a calibration curve prepared for gallic acid and expressed in mg/g dry weight.

#### 4.2.4. Flavonoids Determination

Total flavonoids were determined using the spectrophotometric method according to Czapski and Szwejda [82]. First, 0.5 mL of plant extract was mixed with 1.25 mL of distilled water, then 0.075 mL of 5% sodium nitrite (Sigma-Aldrich, Poznan, Poland) was added. After 6 min, 0.15 mL of 10% aluminum chloride (Sigma-Aldrich, Poznan, Poland) solution was added and left to stand for 5 min. Then, 0.5 mL of 1 M NaOH (Sigma-Aldrich, Poznan, Poland) was added, and the whole was made up to 2.5 mL with water. The absorbance was measured at 510 nm with a UV–Vis spectrophotometer (Hewlett Packard 8453). The flavonoid content was expressed as the catechin equivalent and expressed in mg/g d.w.

### 4.3. Statistical Analysis

Before analysis, the data sets were assessed for normality of distribution and homogeneity of variance (Shapiro–Wilk and Lavene’s tests were applied). A General Linear Model (GLM) with normal distribution and identity link functions was used to investigate the factors affecting the level of phenolic acids and flavonoids in *V. opulus*. The models included plant phenolics level as the response variable and place (garden variety/wild plants) and survey number (7 surveys per each season from end of April to beginning of July) and air temperature as fixed factors.

Analysis of variance (one-way ANOVA) was performed to examine the differences in content of phenolic acids and flavonoids in *V. opulus* tissues. Post hoc Tukey’s test was employed. Data were calculated as the mean of the least twelve independent replicates. All statistical analyses were performed in Statistica version 10.0 (Statsoft Inc., Kraków, Poland). *p* < 0.05 was considered statistically significant.

## 5. Conclusions

In conclusion, this research has investigated the chemical composition of naturally cultivated and wild *V. opulus*. This is the first report on the phenolic acids and flavonoids of *V. opulus* from two places and the effect of temperature and place of occurrence on their content. Based on the results from this study, we conclude that *V. opulus*, both growing wild and in gardens, could be an important source of phenolic acids and flavonoids. The results demonstrated the differences in the bioactive compounds content in *V. opulus* leaves, but the composition was the same. Our results indicate that temperature affects flavonoids and phenol acids content, which is important in the light of global warming, which is readily observed. It was also demonstrated that there is a significant effect of both the temperature and the place of plant growth on phenolic compounds content. Our results indicate that the leaves of *V. opulus*, both growing wild and in gardens, have commercial potential due to their high phenolic acids and flavonoids contents. Although *V. opulus* may have some potential for the human, further investigation on these chemicals, their precise modes of activity and their biological effects, are needed due to the inherent structural diversity of phenolic acids and flavonoids and their impact on the human.

## Figures and Tables

**Table 1:** The effects of temperature, place, survey number and interaction between studied parameters on level of total phenols and flavonoids in *V. opulus* (general linear models GLM, normal error distribution).

Parameter	F_21,146_	*p*
Total phenols		
Temperature	2.21	0.140
Place	6.04	0.003
Survey number	45.67	<0.001
Interaction	7.96	<0.001
Flavonoids		
Temperature	0.20	0.657
Place	0.00	0.999
Survey number	4.75	<0.001
Interaction	9.66	<0.001

**Table 2:** The effects of temperature, place, survey number and interaction between studied parameters on level of hydroxybenzoic acids in *V. opulus* (GLM, normal error distribution).

Parameter	F_4,19_	*p*
Hydroxybenzoic acids		
gallic		
Temperature	2.44	0.134
Place	0.36	0.558
Survey number	0.22	0.647
Interaction	2.38	0.139
p-hydroxybenzoic		
Temperature	1.20	0.288
Place	5.91	0.025
Survey number	1.72	0.205
Interaction	1.22	0.283
syringic		
Temperature	2.75	0.114
Place	1.82	0.193
Survey number	2.32	0.144
Interaction	2.95	0.102
salicylic		
Temperature	1.64	0.215
Place	0.02	0.899
Survey number	1.89	0.184
Interaction	11.15	0.003
benzoic		
Temperature	0.65	0.431
Place	9.69	0.006
Survey number	0.29	0.598
Interaction	0.80	0.382

**Table 3:** The effects of temperature, place, survey number and interaction between studied parameters on level of hydroxycinnamic acids in *V. opulus* (GLM, normal error distribution).

Parameter	F_4,19_	*p*
Hydroxycinnamic acids		
chlorogenic		
Temperature	5.65	0.028
Place	0.09	0.764
Survey number	0.79	0.387
Interaction	20.65	<0.001
caffeic		
Temperature	0.43	0.521
Place	8.42	0.009
Survey number	0.64	0.433
Interaction	0.23	0.634
p-coumaric		
Temperature	0.01	0.941
Place	5.29	0.033
Survey number	2.18	0.156
Interaction	0.00	0.967
ferulic		
Temperature	5.37	0.032
Place	11.74	0.003
Survey number	1.53	0.232
Interaction	11.48	0.003
o-coumaric		
Temperature	2.67	0.118
Place	6.08	0.023
Survey number	2.48	0.132
Interaction	0.04	0.843
t-cinnamic		
Temperature	0.85	0.368
Place	0.73	0.404
Survey number	1.78	0.198
Interaction	1.38	0.255

**Table 4:** The effects of temperature, place, survey number and interaction between studied parameters on level of flavanols in *V. opulus* (GLM, normal error distribution).

Parameter	F_4,19_	*p*
Flavanols		
(+)-catechin		
Temperature	3.87	0.064
Place	0.00	0.946
Survey number	7.70	0.012
Interaction	6.33	0.021
(−)-epicatechin		
Temperature	3.21	0.089
Place	231.69	<0.001
Survey number	59.41	<0.001
Interaction	490.67	<0.001

**Table 5:** The effects of temperature, place, survey number and interaction between studied parameters on level of flavonols in *V. opulus* (GLM, normal error distribution).

Parameter	F_4,19_	*p*
Flavonols		
quercetin		
Temperature	0.83	0.372
Place	17.51	<0.001
Survey number	11.51	0.003
Interaction	300.37	<0.001
rutin		
Temperature	1.03	0.322
Place	276.26	<0.001
Survey number	0.83	0.374
Interaction	178.18	<0.001
kaempferol		
Temperature	0.00	0.973
Place	2.94	0.103
Survey number	1.44	0.245
Interaction	57.35	<0.001
myricetin		
Temperature	1.04	0.321
Place	44.97	<0.001
Survey number	0.51	0.482
Interaction	16.24	0.001

**Table 6:** The effects of temperature, place, survey number and interaction between studied parameters on level of flavones in *V. opulus* (GLM, normal error distribution).

Parameter	F4,19	p
Flavones		
luteolin		
Temperature	2.96	0.101
Place	1.85	0.189
Survey number	4.04	0.059
Interaction	0.00	0.960
apigenin		
Temperature	4.53	0.047
Place	0.04	0.843
Survey number	4.36	0.051
Interaction	1.29	0.269
chrysin		
Temperature	12.61	0.002
Place	19.79	0.000
Survey number	16.27	0.001
Interaction	22.86	<0.001

## Data Availability

Not applicable.

## Sample Availability

Samples are available from the corresponding author upon reasonable request.
